# ViennaNGS: A toolbox for building efficient next- generation sequencing analysis pipelines

**DOI:** 10.12688/f1000research.6157.2

**Published:** 2015-07-20

**Authors:** Michael T. Wolfinger, Jörg Fallmann, Florian Eggenhofer, Fabian Amman

**Affiliations:** 1 Institute for Theoretical Chemistry, University of Vienna, Währingerstraße 17, A-1090, Vienna, Austria; 2 Center for Integrative Bioinformatics Vienna, Max F. Perutz Laboratories, University of Vienna, Medical University of Vienna, Dr. Bohr-Gasse 9, A-1030 Vienna, Austria; 3 Department of Biochemistry and Molecular Cell Biology, Max F. Perutz Laboratories, University of Vienna, Dr. Bohr-Gasse 9, A-1030 Vienna, Austria; 4 Department of Chromosome Biology, Max F. Perutz Laboratories, University of Vienna, Medical University of Vienna, Dr. Bohr-Gasse 9, A-1030 Vienna, Austria

**Keywords:** Perl, next generation sequencing, RNA-seq, read mapping, pipelines

## Abstract

Recent achievements in next-generation sequencing (NGS) technologies lead to a high demand for reuseable software components to easily compile customized analysis workflows for big genomics data. We present ViennaNGS, an integrated collection of Perl modules focused on building efficient pipelines for NGS data processing. It comes with functionality for extracting and converting features from common NGS file formats, computation and evaluation of read mapping statistics, as well as normalization of RNA abundance. Moreover, ViennaNGS provides software components for identification and characterization of splice junctions from RNA-seq data, parsing and condensing sequence motif data, automated construction of Assembly and Track Hubs for the UCSC genome browser, as well as wrapper routines for a set of commonly used NGS command line tools.

## Introduction

Next-generation sequencing (NGS) technologies have influenced both our understanding of genomic landscapes as well as our attitude towards handling big biological data. Emerging functional genomics methods based on high-throughput sequencing allow investigation of highly specialized and complex scientific questions, which continuously poses challenges in the design of analysis strategies. Moreover, the demand for efficient data analysis methods has dramatically increased. While a typical NGS analysis workflow is built on a cascade of routine tasks, individual steps are often specific for a certain assay, e.g. depend on a particular sequencing protocol.

Here, we present ViennaNGS, a Perl distribution that integrates high-level routines and wrapper functions for common NGS processing tasks. ViennaNGS provides tools and functionality for the development of custom NGS pipelines, rather than being an established pipeline per se. It comes with a set of utility scripts that serve as reference implementation for most library functions and can readily be applied for specific tasks or integrated as-is into tailor-made pipelines. Moreover, we provide extensive documentation, including a dedicated tutorial that showcases core features of the software and discusses common application scenarios.

A set of NGS analysis pipelines are available for general ^1, 2^, and specialized assays such as de-novo motif discovery ^3^. While these tools mostly cover the elementary steps of an analysis workflow, they often represent custom-tailored solutions that lack flexibility. Web-based approaches like *Galaxy* ^4^ cover a wide portfolio of available applications, however they do not offer enough room for power users who are used to the benefits of the command line.

The recently published *HTSeq* framework ^5^ as well as the *biotoolbox* package provide library modules for processing high-throughput data. While both packages implement NGS analysis functionality in a coherent manner, we encountered use cases that were not covered by these tools.

ViennaNGS is a pure Perl-based alternative to existing approaches, addressing the broad Perl community in bioinformatics. It partly builds on *BioPerl* ^6^ and has been designed in an object-oriented manner based on the *Moose* object framework, thus allowing to write modular code with different libraries that engage with one another. Moose is based in large part on the Perl 6 object system, thererby enabling rapid conversion to Perl 6. While there is ongoing discussion in the BioPerl community regarding possible directions towards a shift to Perl 6, ViennaNGS is, to our knowledge, the first toolbox for NGS data processing that can be regarded ready for Perl 6.

## Motivation

The motivation for this contribution emerged in the course of the research consortium “RNA regulation of the transcriptome” (Austrian Science Fund project F43), which brings together more than a dozen experimental groups with various thematic backgrounds. In the line of this project it turned out that complex tasks in NGS analysis could easily be automated, whereas linking individual steps was very labour-intensive. As such, it became apparent that there is a strong need for modular and reusable software components that can efficiently be assembled into different full-fledged NGS analysis pipelines. Development of the ViennaNGS suite was triggered by two driving forces. On the one hand we wanted to return to the open source community our own contribution, which itself is heavily based and dependent on open source software. On the other hand, beside “open science” we advocate for the concept of “reproducible science” ^7^. Unfortunately, and to some extent surprising, bioinformatics analyses are often not fully reproducible due to inaccessibility of tools (keyword “in-house script”) or software versions used. It is therefore essential to ensure the entire chain of reproducibility from data generation to interpretation in the analysis of biological data.

## Applications

ViennaNGS has been designed to facilitate the process of builing NGS pipelines. To this end, the toolbox comes with several modules and library functions that can easily be combined into custom analysis workflows. We provide step by guides in the form of dedicated tutorials to lead prospective users through the development of basic NGS analysis pipelines.

### Building a pipeline with ViennaNGS

ViennaNGS::Tutorial is a showcase for building custom analysis pipelines and consists of several chapters, each illustrating an example workflow together with a possible solution based on ViennaNGS library functions. Tutorial #0 shows how to deduce both qualitative and quantitative parameters from mapped reads, together with visual data representation. Tutorial #1 exemplifies the detection of sequence motifs in close proximity to gene start loci in order to identify regulatory regions. Tutorial #2 exemplifies the visualization of highly expressed genes together with a 50 nt region upstream of the gene start and Tutorial #3 explains how to construct UCSC genome browser Assembly Hubs. The tutorials are meant to assist prospective users applying ViennaNGS to implement their own full-fledged pipelines. Moreover, we used the tutorials to demonstrate the run time and memory requirement of sample implementations of ViennaNGS in a real world scenario ( Table 1).

**Table 1.** Time and memory requirements of exemplary implementations of the ViennaNGS core modules, as implemented in the ViennaNGS tutorials. Data were collected on a single core of a desktop workstation (Intel ^®^ Core™ i7-4771 CPU @ 3.50GHz; 32GB RAM).

Script	Input	Run time	RAM
Tutorial #0	4GB BAM file	50m 30s	5.1 GB
Tutorial #1	28GB Fasta, 16KB BED, 292KB XML	0m 38s	219 MB
Tutorial #2	4GB BAM, 28GB Fasta, 16KB BED	7m 49s	663 MB
Tutorial #3	5MB BigBed, 4MB BigWig, 4MB BigBed, 3MB BigWig	0m 1s	213MB

### Utilities

The ViennaNGS suite comes with a collection of complementary executable Perl scripts for accomplishing routine tasks often required in NGS data processing. These command line utilities serve as reference implementations of the routines implemented in the library and can readily be used for atomic tasks in NGS data processing. Table 2 lists the utilities and gives a short description of their functionality.

**Table 2.** Overview of the complementary utilities shipped with ViennaNGS. While some of these scripts are re-implementations of functionality available elsewhere, they have been developed primarily as reference implementation of the library routines to help prospective ViennaNGS users getting started quickly with the development of custom pipelines.

Utility	Description
assembly_hub_constructor.pl	Construct Assembly Hubs for UCSC genome browser visualization
bam_quality_stat.pl	Compute mapping/quality statistics along with publication-ready figures
bam_split.pl	Split BAM files by strand
bam_to_bigwig.pl	Produce BigWig coverage profiles from BAM files for visualization
bam_uniq.pl	Filter uniquely and multi mapped reads from BAM files
bed2bedGraph.pl	Convert BED to (strand specific) BedGraph format
extend_bed.pl	Extend genomic intervals in BED format at the 5′, 3′, or both ends
gff2bed.pl	Convert bacterial RefSeq GFF3 annotation to BED12 format
kmer_analysis.pl	Count k-mers of predefined length in FastQ and Fasta files
MEME_xml_motif_extractor.pl	Compute basic statistics from MEME XML output
newUCSCdb.pl	Create a new genome database in a local UCSC genome browser instance
normalize_multicov.pl	Compute normalized expression data in RPKM and TPM from read counts
sj_visualizer.pl	Convert splice junctions in segemehl BED6 splice junction format to BED12
splice_site_summary.pl	Identify and characterize splice junctions from RNA-seq data
track_hub_constructor.pl	Construct Track Hubs for UCSC genome browser visualization
trim_fastq.pl	Trim sequence and quality fields in FastQ format

## Methods

**Figure 1.**
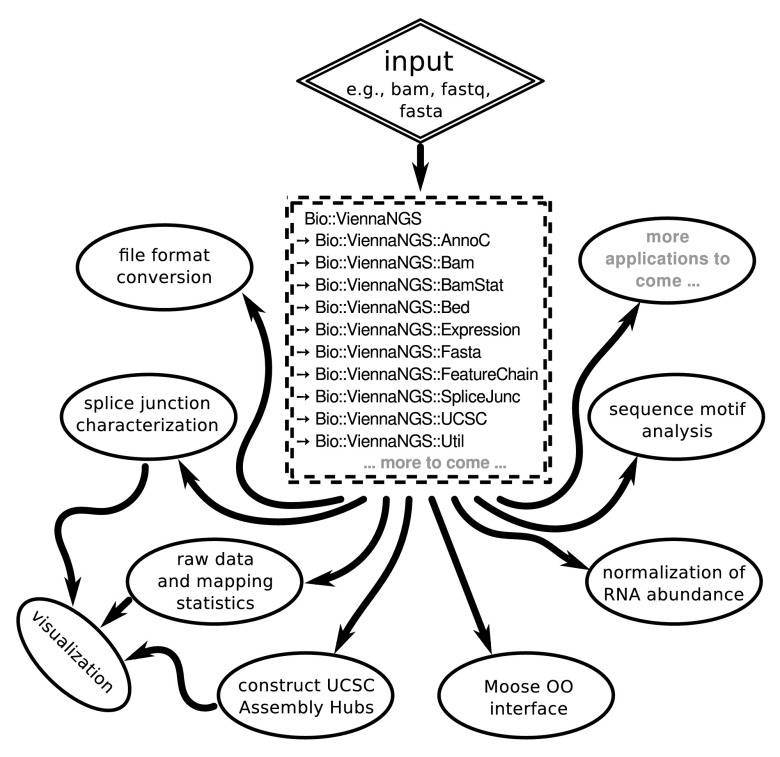
Schematic overview of ViennaNGS components. Core modules can be combined within a data analysis script in a flexible manner to meet individual analysis objectives and experimental setup.

The major design consideration for the ViennaNGS toolbox was to make available modular and reuseable code for NGS processing in a popular scripting language. We therefore implemented thematically related functionality in different Perl modules under the Bio namespace ( Figure 1).

Our focus is on consistent versioning, facilitated through Github hosting. In addition, ViennaNGS releases are available via the Comprehensive Perl Architecture Network (CPAN), thereby enabling users to get back to previous versions at any time in order to reenact conclusions drawn from shared biological data.

ViennaNGS has been designed to close gaps in established analysis workflows by covering a wide range of processing steps from raw data to data visualization. In the following we introduce individual ViennaNGS components and describe their main functionality.

### BAM handling and filtering

Once mapped to a reference genome, NGS data is typically stored in the widely used SAM/BAM file format. BAM is a binary format, which can easily be converted into text-based SAM format via *samtools* ^8^ for downstream analysis. However, modern NGS assays produce hundreds of millions of reads per sample, hence SAM files tend to become excessively large and can have a size of several hundred gigabytes. Given that storage resources are always limited, strategies to efficiently retrieve mapping information from BAM format are an asset. To accomodate that, we provide functionality for querying global mapping statistics and extracting specific alignment information from BAM files directly.

ViennaNGS::BamStat extracts both qualitative and quantitative information from BAM files, i.e. the amount of total alignments, aligned reads, as well as uniquely and multi mapped reads. Numbers are reported individually for single-end reads, paired-end fragments and pairs missing a mate. Quality-wise ViennaNGS::BamStat collects data on edit distance in the alignments, fraction of clipped bases, fraction of matched bases, and quality scores for entire alignments. Subsequently, ViennaNGS::BamStatSummary compares different samples in BAM format and illustrates results graphically. Summary information is made available in CSV format to facilitate downstream processing.

Efficient filtering of BAM files is among the most common tasks in NGS analysis pipelines. Building on the Bio-SamTools distribution, ViennaNGS::Bam provides a set of convenience routines for rapid handling of BAM files, including filters for unique and multiple alignments as well as functionality for splitting BAM files by strand, thereby creating two strand-specific BAM files. Results can optionally be converted to BedGraph or BigWig formats for visualization purposes.

### Genomic annotation

Proper handling of genomic intervals is essential for NGS analysis pipelines. Several feature annotation formats have gained acceptance in the scientific community, including BED, GTF, GFF, etc., each having its particular benefits and drawbacks. While annotation for a certain organism is often only available in a specific format, interconversion among these formats can be regarded a routine task, and a pipeline should be capable of processing as many formats as possible.

**Figure 2.**
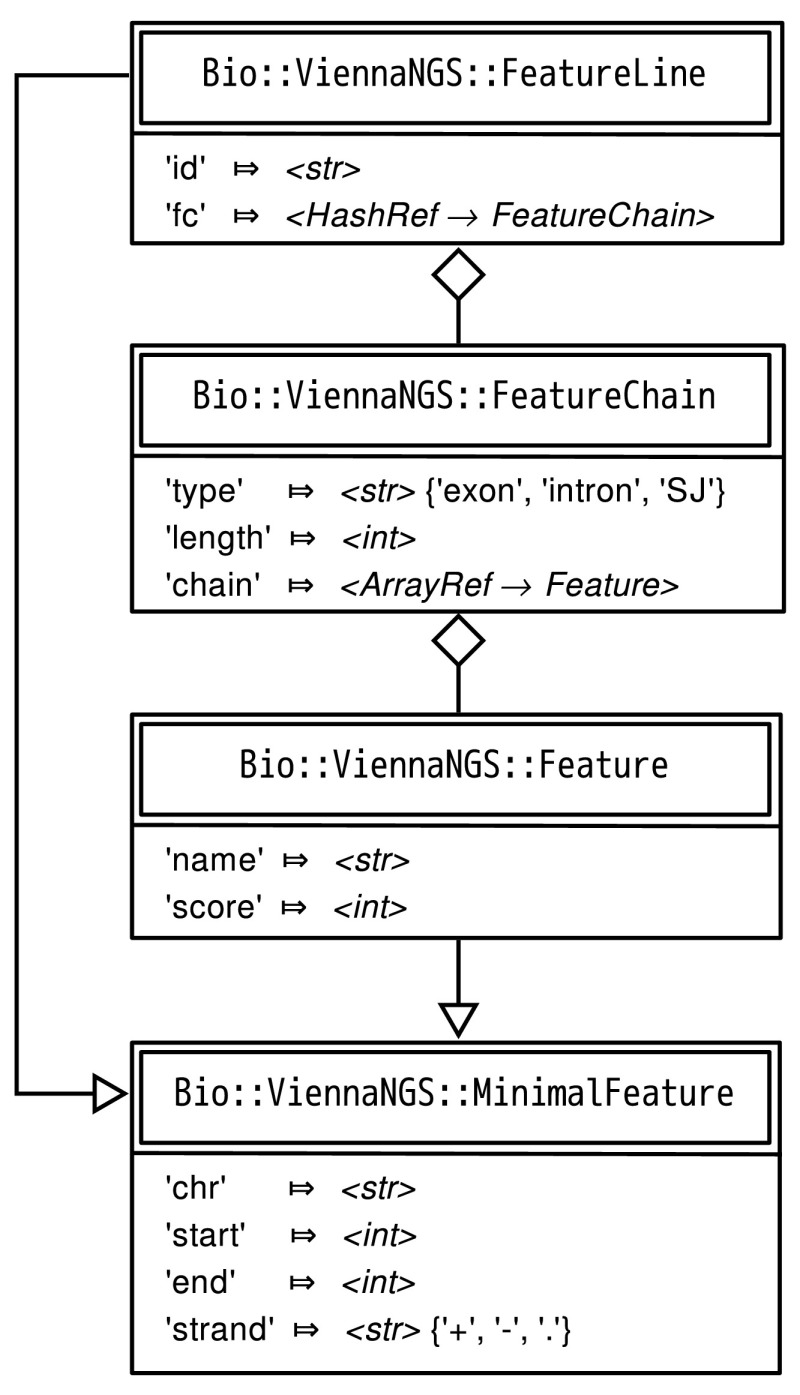
Class diagram illustrating the relations among generic Moose classes which are used as abstract representations of genomic intervals (only attributes are shown).

We address this issue at different levels. On the one hand, we provide ViennaNGS::AnnoC, a lightweight annotation converter for non-spliced genomic intervals, which can be regarded a simple yet powerful solution for conversion of bacterial annotation data. On the other hand we have developed an abstract representation of genomic features via generic *Moose*-based classes, which provide functionality for efficient manipulation of BED4, BED6, BED12 and GTF/GFF elements, respectively, and allow for BED format conversion facilitated by ViennaNGS::Bed. ViennaNGS::MinimalFeature represents an elementary genomic interval, characterized by chromosome, start, end and strand. ViennaNGS::Feature extends ViennaNGS::MinimalFeature by two attributes, name and score, thereby creating a representation of a single BED6 element. ViennaNGS::FeatureChain pools a set of ViennaNGS::Feature objects via an array reference. All intervals of interest can be covered by a ViennaNGS::FeatureLine object, which holds a hash of references to ViennaNGS::FeatureChain objects ( Figure 2).

**Figure 3.**
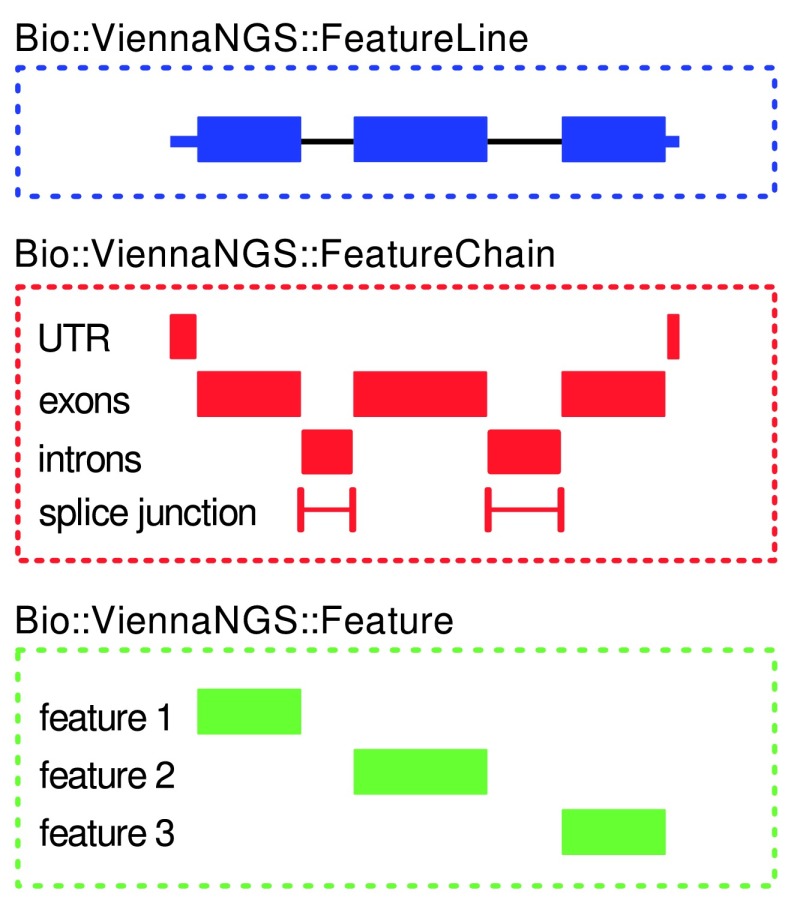
Schematic representation of genomic interval classes in terms of their corresponding feature annotation. Simple intervals (“features”) are characterized by ViennaNGS::Feature objects (bottom box). At the next level, ViennaNGS::FeatureChain bundles these, thereby maintaining individual annotation chains for e.g. UTRs, exons, introns, splice junctions, etc. (middle box). The topmost level is given by ViennaNGS::FeatureLine objects, representing individual transcripts.

This framework can handle annotation data by providing abstract data representations of genomic intervals such as exons, introns, splice junctions etc. It allows for efficient description and manipulation of genomic features up to the level of transcripts ( Figure 3). Conversely, it is highly generic and can be extended to hierarchically higher levels such as genes composed of different transcript isoforms or clusters of paralogous genes.

### Visualization

Another cornerstone of NGS analysis pipelines is graphical representation of mapped sequencing data. In this context a standard application is visualization of ChIPseq peaks or RNA-seq coverage profiles. The latter are typically encoded in Wiggle format, or its indexed binary variant, BigWig, which can readily be displayed within a genome browser. In the same line, genomic annotation and intervals are often made available in BigBed format, an indexed binary version of BED. ViennaNGS::Util comes with wrapper routines for automated conversion from common formats like BAM to BigWig or BED to BigBed via third-party utilities ^9^. In addition, we have implemented interfaces for a selection of *BEDtools* ^10^ components as well as a collection of auxiliary routines. The UCSC genome browser allows to display potentially large genomic data sets, that are hosted at web-accessible locations by means of Track Hubs ^11^. On a more general basis this even works for custom organisms that are not supported by default through the UCSC genome browser, via Assembly Hubs. A typical use case is visualization of genomic annotation, RNA-seq coverage profiles and ChIPseq peaks for *Arabidopsis thaliana* (which is not available through the generic UCSC browser) via a locally hosted Assembly Hub. ViennaNGS::UCSC provides all relevant routines for automatic construction of Assembly and Track Hubs from genomic sequence and/or annotation. Besides automated Assembly and Track Hub generation, we support deployment of custom organism databases in local mirrors of the UCSC genome browser.

### Gene expression and normalization

RNA-seq has become a standard approach for gene and transcript quantification by means of measuring the relative amount of RNA present in a certain sample or under a specific condition, thus ideally providing a good estimate for the relative molar concentration of RNA species. Simple “count-based” quantification models assume that the total number of reads mapping to a region can be used as a proxy for RNA abundance ^12^. A good measure for transcript abundance is ideally as closely proportional to the relative molar concentration of a RNA species as possible. Various measures have been proposed, one of the most prominent being RPKM (reads per kilobase per million). It accounts for different transcript lengths and sequencing depth by normalizing by the number of reads in a specific sample, divided by 10 ^6^. It has, however, been shown that RPKM is not appropriate for measuring the relative molar concentration of a RNA species due to normalization by the total number of reads ^13, 14^.

Alternative measures that overcome this shortcoming have been suggested, e.g. TPM (transcript per million), where a proxy for the total number of transcript samples considering the sequencing reads per gene is used for normalization, rather than the total number of mapped reads. We provide routines for the computation of RPKM and TPM values for genomic intervals from raw read counts within ViennaNGS::Expression.

### Characterization of splice junctions

ViennaNGS::SpliceJunc addresses a more specific problem, namely characterization of splice junctions which is becoming increasingly relevant for understanding alternative splicing events. This module provides code for identification and characterization of splice junctions from short read mappers. It can detect novel splice junctions in RNA-seq data and generate visualization files. While we have focused on processing the output of *segemehl* ^15, 16^, the module can easily be extended for other splice-aware split read mappers.

### Documentation

The ViennaNGS suite comes with extensive documentation based on Perl’s POD system, thereby providing a single documentation base which is available through different channels, e.g. on the command line via the *perldoc* utility or on the Web via CPAN.

### Testing

In the development process of the ViennaNGS suite special emphasis has been placed on code integrity, thereby ensuring that the software produces correct results as novel features are added and the code base is maintained. To achieve that, we make use of the Perl testing framework, which allows to build automated tests that are run at installation time and highlight any issues with code or third party dependencies. Furthermore this includes comparison of MD5 sums for output files produced by ViennaNGS routines, thereby enabling consistency and reproducibility of biological results.

## Use cases

We have successfully applied components of ViennaNGS in the course of an ongoing, large scale collaboration project focusing on RNA regulation. It has been used with different genomics assays in a wide range of biological systems, including human, plants and bacteria. While we have primarily applied ViennaNGS in combination with the short read aligner *segemehl* ^15, 16^, e.g. in a study addressing ribosome associated mRNA degradation in *Drosophila* ^17^, it has also been used recently with *Tophat* ^18^ output in a large scale transcriptome study of Ebola and Marburg virus infection in human and bat cells (Hölzer *et al.*, unpublished data).

## Discussion

ViennaNGS is a comprehensive software library for rapid development of custom NGS analysis pipelines. An aspect that is becoming increasingly relevant in scientific computation is parallelization. While we have focused on code convenience, feature richness and easy extensibility, custom ViennaNGS-based pipelines can potentially be implemented in a parallel manner by the end user, e.g. through the Perl threads functionality. An example would be to process and filter a set of BAM files in parallel, provided sufficient IO resources are available.

ViennaNGS is actively developed and its code base is constantly maintained and expanded. We will provide a generic, Moose based annotation converter that builds on and extends the feature annotation classes in the future. In addition, we will incorporate functionality for manipulation and storage of sequence variants, such as SNPs, editing and modification events. ViennaNGS will also be used for automated UCSC genome browser integration in an upcoming version of TSSAR ^19^, a recently published approach for characterization of transcription start sites from dRNA-seq data. Moreover, we will provide Bio::HubFactory, a ViennaNGS-based Web Service for automatic generation of UCSC genome browser Assembly Hubs for all RefSeq bacteria.

ViennaNGS is an open platform for building specialized NGS pipelines, which fills a niche by providing functionality that is, to our knowledge, not available elsewhere. In this line, we would like to encourage the scientific community to contribute novel features and patches via Github.

## Data availability

Input data for the ViennaNGS tutorial is available from http://rna.tbi.univie.ac.at/ViennaNGS.

## Software availability

The ViennaNGS distribution is available through the Comprehensive Perl Architecture Network (CPAN) and at GitHub.

1. http://search.cpan.org/dist/Bio-ViennaNGS
2. https://github.com/mtw/Bio-ViennaNGS
3. Software license: The Perl 5 License

## Third party dependencies

The ViennaNGS toolbox depends on a set of third-party tools and libraries which are required for specific filtering and file format conversion tasks as well as for building internally used Perl modules:

- BEDtools >= 2.17 ^10^
- bedGraphToBigWig, fetchChromSizes and faToTwoBit from the UCSC Genome Browser applications ^9^
- the R Statistics software ^20^
- samtools <= v0.1.19 ^13^ for building Bio::DB::Sam. Please note that more recent HTSlib-based versions of samtools will not work with Bio::DB::Sam

## References

[1] Förstner KU Vogel J Sharma CM: READemption-a tool for the computational analysis of deep-sequencing-based transcriptome data. *Bioinformatics.* 2014;30(23):3421–3. 10.1093/bioinformatics/btu533 25123900

[2] Breese MR Liu Y: NGSUtils: a software suite for analyzing and manipulating next-generation sequencing datasets. *Bioinformatics.* 2013;29(4):494–6. 10.1093/bioinformatics/bts731 23314324 PMC3570212

[3] Heinz S Benner C Spann N: Simple combinations of lineage-determining transcription factors prime *cis*-regulatory elements required for macrophage and B cell identities. *Mol Cell.* 2010;38(4):576–89. 10.1016/j.molcel.2010.05.004 20513432 PMC2898526

[4] Goecks J Nekrutenko A Taylor J: Galaxy: a comprehensive approach for supporting accessible, reproducible, and transparent computational research in the life sciences. *Genome Biol.* 2010;11(8):R86. 10.1186/gb-2010-11-8-r86 20738864 PMC2945788

[5] Anders S Pyl PT Huber W: HTSeq--a Python framework to work with high-throughput sequencing data. *Bioinformatics.* 2015;31(2):166–9. 10.1093/bioinformatics/btu638 25260700 PMC4287950

[6] Stajich JE Block D Boulez K: The Bioperl toolkit: Perl modules for the life sciences. *Genome Res.* 2002;12(10):1611–8. 10.1101/gr.361602 12368254 PMC187536

[7] Stodden V Leisch F Peng RD: Implementing Reproducible Research. CRC Press,2014 Reference Source

[8] Li H Handsaker B Wysoker A: The Sequence Alignment/Map format and SAMtools. *Bioinformatics.* 2009;25(16):2078–9. 10.1093/bioinformatics/btp352 19505943 PMC2723002

[9] Kent WJ Zweig AS Barber G: BigWig and BigBed: enabling browsing of large distributed datasets. *Bioinformatics.* 2010;26(17):2204–7. 10.1093/bioinformatics/btq351 20639541 PMC2922891

[10] Quinlan AR Hall IM: BEDTools: a flexible suite of utilities for comparing genomic features. *Bioinformatics.* 2010;26(6):841–2. 10.1093/bioinformatics/btq033 20110278 PMC2832824

[11] Raney BJ Dreszer TR Barber GP: Track data hubs enable visualization of user-defined genome-wide annotations on the UCSC Genome Browser. *Bioinformatics.* 2014;30(7):1003–1005. 10.1093/bioinformatics/btt637 24227676 PMC3967101

[12] Pachter L: Models for transcript quantification from RNA-Seq. *arXiv preprint arXiv: 1104.3889.* 2011 Reference Source

[13] Li B Ruotti V Stewart RM: RNA-Seq gene expression estimation with read mapping uncertainty. *Bioinformatics.* 2010;26(4):493–500. 10.1093/bioinformatics/btp692 20022975 PMC2820677

[14] Wagner GP Kin K Lynch VJ: Measurement of mRNA abundance using RNA-seq data: RPKM measure is inconsistent among samples. *Theory Biosci.* 2012;131(4):281–285. 10.1007/s12064-012-0162-3 22872506

[15] Hoffmann S Otto C Kurtz S: Fast mapping of short sequences with mismatches, insertions and deletions using index structures. *PLoS Comput Biol.* 2009;5(9):e1000502. 10.1371/journal.pcbi.1000502 19750212 PMC2730575

[16] Hoffmann S Otto C Doose G: A multi-split mapping algorithm for circular RNA splicing, trans-splicing, and fusion detection. *Genome Biol.* 2014;15(2):R34. 10.1186/gb-2014-15-2-r34 24512684 PMC4056463

[17] Antic S Wolfinger MT Skucha A: General and MicroRNA-Mediated mRNA Degradation Occurs on Ribosome Complexes in *Drosophila* Cells. *Mol Cell Biol.* 2015;35(13):2309–20. 10.1128/MCB.01346-14 25918245 PMC4456442

[18] Trapnell C Pachter L Salzberg SL: TopHat: discovering splice junctions with RNA-Seq. *Bioinformatics.* 2009;25(9):1105–1111. 10.1093/bioinformatics/btp120 19289445 PMC2672628

[19] Amman F Wolfinger MT Lorenz R: TSSAR: TSS annotation regime for dRNA-seq data. *BMC Bioinformatics.* 2014;15:89. 10.1186/1471-2105-15-89 24674136 PMC4098767

[20] R Core Team. R: A Language and Environment for Statistical Computing. R Foundation for Statistical Computing, Vienna, Austria,2014 Reference Source

