# Supplementary material for: ViennaNGS: A toolbox for building efficient next- generation sequencing analysis pipelines
Source: F1000Res. 2015 Jul 20;4:50. Originally published 2015 Feb 20. [Version 2] doi: 10.12688/f1000research.6157.2 (PMC4513691; doi:10.12688/f1000research.6157.2)
Supplement: Peer review history

# Peer review: Referee response for version 2

**DOI:** 10.5256/f1000research.7298.r9558

**Published:** 2015-07-21

**Authors:** Björn Voß

**Affiliations:** 1 Faculty of Biology, University of Freiburg, Freiburg, Germany

The authors have significantly improved their manuscript and satisfactorily replied to my comments, such that I feel happy to approve this version of the manuscript.

I have read this submission. I believe that I have an appropriate level of expertise to confirm that it is of an acceptable scientific standard.

# Peer review: Referee response for version 1

**DOI:** 10.5256/f1000research.6600.r8396

**Published:** 2015-04-24

**Authors:** Björn Voß

**Affiliations:** 1 Faculty of Biology, University of Freiburg, Freiburg, Germany

In their manuscript about ViennaNGS the authors describe a set of perl modules and scripts that is useful to build pipelines for NGS data analysis. A key motivation for this is to promote reproducible science, especially with respect to medium-level users, who often create "in-house scripts" for data analysis, which are rarely publicly available. This target community distinguishes ViennaNGS from related approaches, such as Galaxy. The contribution is, thus, relevant and has the potential to serve as a basis for future developments in NGS analysis pipelines. I tested the tutorials and some of the utility scripts and they worked fine. Nevertheless, I think the authors need to clarify some issues and can improve the presentation of their work.

Major Comments:

- The authors should point out clearly, what distinguishes ViennaNGS from other suites. In the end, they need to convince people to use ViennaNGS. For that  it would be helpful to clearly state what is hard or even impossible to implement in one of the other systems (galaxy, HTSeq, ...) at best with real world examples.
- As stated in the title the aim of ViennaNGS is to ease the process of building NGS analysis pipelines. Unfortunately, exactly this aspect is more or less not mentioned in the main text. It would be interesting to know, especially for the data analysts with scripting experience, how such a pipeline looks like and why it is easier to build using ViennaNGS.
- I do not quite understand the explicit discussion of TPM and RPKM. The differences are extensively discussed in Wagner *et al. *(2012), which the authors can refer to.
- Similarly, the description of the accompanying utilities in Table 1 is of minor interest. I would suggest to mention them when the corresponding functionality is described in the main text, e.g., assembly_hub_constructor.pl in the paragraph on Visualization. Furthermore, the authors can explain one tool in detail to show how ViennaNGS pipelines are implemented.
- BioPerl already provides modules to handle Annotation Features (Bio::SeqFeature), which at first glance seem to provide the same functionality as the ViennaNGS feature annotation classes. Why is there a need for an own class?

Minor Comments:

- An aspect that is becoming more and more important is parallelization. The authors should describe the possibilities of ViennaNGS to be used in cluster or massively parallel environments.
- The authors should make clear that for some/many tasks they use external tools, such as bedtools2, samtools and tools offered by the UCSC and that the user has to install them on its own. Of course, this is the same as for galaxy and others.
- I was wondering if ViennaNGS or its pipelines may be integrated into Galaxy. In this way the systems would complement and benefit from each other.
- At the end of the discussion the authors could provide actual functionalities that they are planning to integrate in the near future. This is interesting for potential users who are missing certain functionalities in the current release. On example is quality control of the raw sequencing data.
- P.5, Software availability: "at and" --> "and at"

I have read this submission. I believe that I have an appropriate level of expertise to confirm that it is of an acceptable scientific standard, however I have significant reservations, as outlined above.

# Peer review: Referee response for version 1

**DOI:** 10.5256/f1000research.6600.r8057

**Published:** 2015-04-23

**Authors:** Brad Chapman

**Affiliations:** 1 Department of Biostatistics, Harvard Public School of Health, Boston, MA, USA

The authors describe ViennaNGS, a set of Perl modules and scripts to provide RNA-seq analysis and visualization via UCSC integration. The code is nicely written, open source and easy to install via CPAN with cpanminus. Additionally, the documentation is excellent and contains both high level material in the form of blog posts as well as detailed source code descriptions. In reading the paper I found a few areas that would help improve reader's understanding of the toolbox:

- Please include additional information about what is unique about ViennaNGS in the introduction. Currently it reads generally and is more about pointing about flaws in other software without saying what ViennaNGS provides. The motivation provides much of this text but it seems out of order relative to the introductory material.
- Please provide benchmarks on your BAM manipulation tools relative other common tools. I don't think this needs to be extensive, but providing a summary of how they perform on a 100Gb 30x whole human genome sequence would be helpful. For filtering comparisons, I suggest comparing with samtools or sambamba (https://github.com/lomereiter/sambamba). For quality control, comparisons to QualiMap (http://qualimap.bioinfo.cipf.es/) or bamtools (https://github.com/pezmaster31/bamtools) would be helpful.
- Similarly, it would be great to have benchamrking on annotation and BED manipulation tools in ViennaNGS. How does the functionality and timing compare with bedtools? You require and use bedtools for visualization, and it would be useful to clarify benefits and tradeoffs to using ViennaNGS versus interfacing directly with bedtools.
- How do you handle testing and validation of ViennaNGS tools and pipelines? I saw new tests for UCSC integration coming in during review, which is great. It would be nice to understand the process by which you ensure new development improves (or at least doesn't degrade) the biological results.

I have read this submission. I believe that I have an appropriate level of expertise to confirm that it is of an acceptable scientific standard, however I have significant reservations, as outlined above.

# Peer review: Referee response for version 1

**DOI:** 10.5256/f1000research.6600.r8365

**Published:** 2015-04-17

**Authors:** Angelika Merkel

**Affiliations:** 1 Centro Nacional de Análisis Genómico, Parc Científic de Barcelona, Barcelona, Spain

The authors present a useful and relevant toolbox for the analysis of NGS data. Its modular design allows for flexibility in the analysis, and the utilization of track hubs for easy exchange of data as well as visualization with popular tools. A nice implementation is the ability to adapt genome annotations of various formats.

Still, I feel the description of the software is rather too general and could be improved.

Major Comments:

The article lacks any benchmarking or presentation of an example analysis, making it difficult to put the software's performance in perspective with any of the  other numerous tools already available. Important for NGS data analysis are specifications for the usage of computational resources (RAM, number of CPUs, processing time, space requirements) and how those scale up with the size of the data set (=number and size of data sets) or type of NGS data (genomic, RNAseq, ChIPseq, Bisulfite-Seq) - all of which are not mentioned. Similarly, the authors do not make any statement on the possibility of parallelization or adaption to cluster infrastructures.

Minor comments:

Although, truly RPKM has been shown to be inappropriate for measuring the relative molar concentration of a RNA species due to normalization by the total number of reads, it is still widely used. Computing RPMK values as well (optionally) as TPM would allow for comparison with other pipelines.

I have read this submission. I believe that I have an appropriate level of expertise to confirm that it is of an acceptable scientific standard, however I have significant reservations, as outlined above.
